# Supplementary material for: Advance Care Planning in Neurodegenerative Disorders: A Scoping Review
Source: Int J Environ Res Public Health. 2022 Jan 12;19(2):803. doi: 10.3390/ijerph19020803 (PMC8775509; doi:10.3390/ijerph19020803)
Supplement: Supplementary file 1 [file ijerph-19-00803-s001.zip › Fig. S2_Risk of bias summary ACP.pdf]

Figure S2. Risk of bias summary: review authors' judgements about each risk of bias item for each included study.

	Random sequence generation (selection bias)	Allocation concealment (selection bias)	Blinding of participants and personnel (performance bias)	Blinding of outcome assessment (detection bias)	Incomplete outcome data (attrition bias)	Selective reporting (reporting bias)	Other bias
Bonner 2020	+	?	?	?	−	+	+
Brazil 2018	+	−	?	?	+	+	?
Goossens 2020a	+	?	+	+	?	+	?
Hanson 2017	?	?	?	+	+	+	+
Mitchell 2018	+	+	+	+	+	+	?
Mitchell 2020	+	?	?	+	+	+	+
Saevareid 2019	?	?	?	−	+	+	?
Sampson 2011	+	?	?	+	?	?	+
Song 2019	+	+	?	+	?	+	?
Tilburgs 2020a	+	+	+	+	?	+	+
