# Supplementary material for: Advance Care Planning in Neurodegenerative Disorders: A Scoping Review
Source: Int J Environ Res Public Health. 2022 Jan 12;19(2):803. doi: 10.3390/ijerph19020803 (PMC8775509; doi:10.3390/ijerph19020803)
Supplement: Supplementary file 1 [file ijerph-19-00803-s001.zip › File S2.pdf]

File S2: PubMed search strategy

Research question: Advance care planning in neurodegenerative disorders

Search dates: inception – 28th December 2020

"Advance Care Planning"[Mesh] OR "advance care planning" OR "advance medical planning" OR "advance health care planning" OR advance directive* OR "Advance Directives"[Mesh] OR "Terminal Care"[Mesh] OR "terminal care" OR "Living Wills"[Mesh] OR living will* OR "advanced care planning" OR "Treatment Refusal"[Mesh] OR "Withholding Treatment"[Mesh] OR "withholding treatment" OR "advance decision" OR ACP OR treatment refus* OR treatment withhold* OR treatment withdraw* OR SDM OR shared decision* OR health care wish* OR "wish for end-of-life care" OR "wishes for end-of-life care" OR "wishes for end of life care" OR "wish for end of life care" OR health care preference* OR “preference for end-of-life care" OR "preferences for end-of-life care" OR "preferences for end of life care" OR "preference for end of life care" OR end-of-life preference* OR end of life preference* OR palliative care plan* OR "desired care" OR end of life decision* OR end-of-life decision* OR advance decision* OR advance care plan* OR goals of care

AND

Progressive neurological diseases OR "Neurology"[Mesh] OR neurol* OR "Nervous System Diseases"[Mesh] OR Nervous System Disease OR Neurologic* Disorders OR Nervous System Disorder* OR "Neurodegenerative Diseases"[Mesh] Neurodegenerative Disease* OR Neurodegenerative Disorder* OR Neurologic Degenerative Condition* OR Degenerative Neurologic Disorder* OR dementia OR "Dementia"[Mesh] OR "Alzheimer Disease"[Mesh] OR Alzheimer's Disease OR Senile Dementia OR Alzheimer dementia OR "Huntington Disease"[Mesh] OR Huntington Chorea OR Huntington's Disease OR "Amyotrophic Lateral Sclerosis"[Mesh] OR Gehrig Disease OR Charcot Disease OR Guam Disease OR Amyotrophic Lateral Sclerosis OR ALS OR "Parkinson Disease"[Mesh] OR Idiopathic Parkinson's Disease OR Lewy Body Parkinson's Disease OR Primary Parkinsonism OR "Parkinson Disease, Secondary"[Mesh] OR secondary Parkinson Disease OR Symptomatic Parkinson Disease OR Atherosclerotic Parkinsonism OR "Multiple Sclerosis"[Mesh] OR Disseminated Sclerosis OR MS OR Multiple Sclerosis OR "Neuromuscular Diseases"[Mesh] OR Neuromuscular Disease OR Foley-Denny-Brown Syndrome OR Benign Fasciculation-Cramp Syndrome* OR Cramp-Fasciculation Syndrome* OR Oppenheims Disease OR Amyotonia Congenita OR "Brain Neoplasms"[Mesh] OR Brain Neoplasm OR brain tumor OR brain cancer
