# Supplementary material for: Advance Care Planning in Neurodegenerative Disorders: A Scoping Review
Source: Int J Environ Res Public Health. 2022 Jan 12;19(2):803. doi: 10.3390/ijerph19020803 (PMC8775509; doi:10.3390/ijerph19020803)
Supplement: Supplementary file 1 [file ijerph-19-00803-s001.zip › Table S1.pdf]

Table S1. Overview of included studies.

First author, publication year[ref. no.]	Neurodegenerative disorder	ACP definition	Country	Design	Sample	Setting	Aim
Abbott et al, 2017[81]	Duchenne muscular dystrophy	EOL/conversations/ decision making	UK	Qualitative study	People with DMD (N=15)	Community	To deepen understanding regarding a range of EOL issues, interventions, practices, and planning; and to focus on views and preferences of men with Duchenne muscular dystrophy.
Ampe et al, 2017[61]	Dementia	Process	Belgium	Quasi-experimental pre-test–post-test study	NH staff: IG (N=19); CG (N=9).	NH	To assess the influence of 'weDECide–Discussing End-of-life Choices' intervention on policy and practice of ACP in NHs. To investigate the NH management and clinical staff's perceived barriers and facilitators for the implementation of SDM in ACP.
Beck et al, 2015[53]	Dementia	Process	UK	Narrative literature review	HPs (N=1305)	Community, NH, inpatients	To provide an up-to-date synthesis of HPs' perspectives on ACP, and of challenges encountered.
Benditt et al, 2001[67]	ALS/MND	Process	US	Commentary	Patients with ALS	n.a.	To devise a disease-specific ACP tool for ALS, focusing on the use of mechanical ventilation.
Bonner et al, 2020[33]	Dementia	Not reported	US	RCT	Caregivers of people with dementia: IG (N=173); CG (N=185)	Community	To assess the efficacy of the Advance Care Treatment Plan intervention, in comparison with attention control.
Brazil et al, 2018[34]	Dementia	Process	UK	RCT	Residents with dementia, including family members and GPs: NH IG, N=12; NH CG: N=12.	NH	To develop a family focused ACP intervention and evaluate its impact in dementia care NHs.
Cheung et al, 2017[82]	Stroke, myopathies, MND, MS, parkinsonism	AD/discussion	China	Observational, retrospective study	Neurology patients (N=25)	Inpatient	To assess EOL care preferences, documentation, and communication in patients with various types of advanced neurological disorders.

First author, publication year[ref. no.]	Neurodegenerative disorder	ACP definition	Country	Design	Sample	Setting	Aim
Cotter et al, 2019[62]	Dementia	Conversations, written document/AD	US	Pre-post study (one arm)	Physicians (N=164)	Inpatient	To increase the rate of ACP conversations and documentation in a dementia specialty practice; and to assess the effectiveness of a brief, educational intervention on physician knowledge, attitudes, and skills; and to evaluate the prevalence of ACP documentation in the electronic medical record.
Cottrell et al, 2020	MS	Process	UK	Realist review	-	Community, NH, inpatients	To develop and revise a theory on ACP engagement for patients with MS, and to identify ways to improve its uptake.
Cutshall et al, 2020[8]	Brain tumors	Conversations	US	Qualitative study	Brain tumor patients (N=21), caregivers (N=5), advocates or organizational members (N=12), and clinicians or researchers (N=14).	Community	To analyze data from the Brain Tumor Stakeholder Twitter community chat, and to identify barriers of brain tumor stakeholders to ACP conversations.
Dassel et al, 2019[52]	Dementia	Process/AD	US	Mixed-method	Phase 1: people with early-stage dementia (N=6), and caregivers (N=11). Phase 2: HPs (N=10); Phase 3: older healthy adults (N=153) and people with early-stage dementia (N=38). Phase 4: caregivers (N=14), healthy adults (N=10), people with early-stage dementia (N=6).	Community, inpatients	To develop a valid and reliable tool which captures the EOL care preferences and values of people with dementia.

First author, publication year[ref. no.]	Neurodegenerative disorder	ACP definition	Country	Design	Sample	Setting	Aim
Fried et al, 2020[45]	Dementia	Process	US	Qualitative study	People with mild cognitive impairment or early dementia (N=28), and caregivers (N=19)	Outpatient	To investigate views on participation in dementia-specific ACP planning among patients with early-stage cognitive impairment and their caregivers. To deepen participants' understanding of the needs for and readiness to plan, and of barriers/facilitators for planning.
Fritz et al, 2020[75]	Brain tumors	Process	The Netherlands	Qualitative study	Focus group: HPs (N=9). Interviews: patient-proxy dyads (N=8), proxies of deceased patients (N=5).	Outpatient	To develop an ACP program specifically for glioblastoma patients. To identify topics which are relevant for patients and their proxies, as well as practical issues and barriers/facilitators to participate in the ACP program.
Geshell et al, 2019[42]	Dementia	Process	US	Integrative literature review	Patients, carers, or HPs ranging from 6 to 661. Patients living with dementia (N=1304)	Community, NH, inpatients, outpatient	To identify and evaluate the literature regarding the perspectives and the experiences of people with dementia on ACP; and to inform current research and practice.
Goossens et al, 2020[35]	Dementia	Process	Belgium	RCT	NH wards (IG, N=34, CG, N=31), including staff members (N=311).	NH	To increase the level of SDM in ACP conversations as well as the perceived competencies, the importance and frequency of SDM in dementia care.
Goossens et al, 2020[51]	Dementia	Process	Belgium	Observational, cross-sectional	HPs (N=311)	NH	To explore how people with dementia and HPs perceive the SDM level during ACP conversations. To assess HPs' perceptions of the importance of SDM, their competence and frequency of utilizing SDM.
Hanson et al, 2017[39]	Dementia	Goals of care	USA	RCT	People with advanced dementia (IG, N=151; CG, N=151), and their family decision makers (N=22)	NH	To investigate whether the goals-of-care intervention improves decision making and quality of communication; to test whether the intervention would improve palliative care for advanced dementia.
Hossler et al, 2011[68]	ALS	Process	USA	Pre-post study (one arm)	People with ALS (N=17)	Outpatient	To evaluate the feasibility of 'Making Your Wishes Known' decision aid for helping patients with ALS engage in ACP.

First author, publication year[ref. no.]	Neurodegenerative disorder	ACP definition	Country	Design	Sample	Setting	Aim
Huang et al, 2020[63]	Dementia	EOL discussion	Taiwan	Pre-post study (one arm)	People with mild cognitive impairment or mild dementia and their family caregivers (N=40 dyads)	Outpatient	To explore the effects of a family-centered ACP information intervention in people with dementi and their carers.
Katwa et al, 2020[60]	Dementia	Process	UK	Pre-post study (one arm)	Care home staff (N=78)	Community	To evaluate the effect of a one-day simulation training for care home staff on ACP.
Keijzer-van Laarhoven et al, 2020[54]	Dementia	EOL discussion	The Netherlands	Meta-review of systematic reviews and primary studies	-	Community, outpatient	To better understand the physicians' moral considerations in long- term care and primary care settings with ethical dilemmas on ACP with people with dementia and their carers.
Konttila et al, 2020[57]	Dementia	EOL discussion	Finland	Observational, retrospective study	Patients with advanced dementia (N=403): Group 1 (N=212), and Group 2 (N=191).	NH	To investigate changes in ACP related to physician treatment orders, symptom prevalence, and possible burdensome interventions of patients who died between 2004–2009 and 2010–2013.
Lai et al, 2019[42]	Dementia	Process	Australia	Systematic integrative review	-	Community, NH	To identify the key factors for engagement in the decision-making process related to the future care of people with dementia.
Lee et al, 2018[56]	Dementia	Process	Canada	Observational, cross-sectional	HPs (N=261)	Outpatient	To assess HPs' desire for more information/education on ACP, capacity for/attitudes toward ACP, and current ACP practices in their clinical practice.
Lee et al, 2020[50]	Dementia	Discussions/process	Canada	Mixed-method	Surveys: HPs (N=12), people with dementia (N=13), and surrogate decision makers (N=16). Interviews: HPs (N=8); people with dementia (N=1), and surrogate decision makers (N=7)	Outpatient	To pilot test an ACP framework tool to be used with people with dementia in primary care-based memory clinics; To describe user/recipient experiences with this framework.

First author, publication year[ref. no.]	Neurodegenerative disorder	ACP definition	Country	Design	Sample	Setting	Aim
Levi et al, 2017[69]	ALS/MND	Not reported	US	Pre-post study (one arm)	Patients with ALS (N=44); HPs (N=15)	Outpatient	To assess the impact of an interactive web-based decision aid on communication between patients with ALS and treating clinicians regarding EOL wishes.
Llewellyn et al, 2018[76]	Brain tumors	Process	UK	Qualitative study	HPs (N=15)	Outpatient	To investigate key social and structural conditions contributing to an observed avoidance of ACP practice by HPs working in neuro-oncology.
Lum et al, 2019[9]	Parkinson's disease	Process	US	Qualitative study	Patients (N=60)	Outpatient	To elicit perspectives from patients and carers on ACP to inform a patient and care partner-centered framework for Parkinson's disease research and clinical practice.
Mitchell et al, 2018[36]	Dementia	Discussions	US	RCT	People with advanced dementia (N=402) and their proxies (IG: N=212; CG: N=190). NHs (N=64: IG N=32; CG N=32.	NH	To test the impact of an ACP video (versus usual care) on documented ADs, level of care preferences, goals-of-care discussions, and burdensome treatments among NH people with advanced dementia.
Mitchell et al, 2020[31]	Dementia	Modifiable factor associated with better palliative care outcome	US	RCT	NHs (IG, N=119; CG, N=241).	NH	To test the effect of an ACP video program on hospital transfers, burdensome treatments, and hospice enrollment among long-stay nursing home residents with and without advanced illness.
Moss et al, 2018[59]	Dementia	EOL planning	US	Observational, cross-sectional	Caregivers (N=65)	Community	To determine the number of formal/informal EOL care plans that existed among a convenience sample of African American older adults with dementia.
Murray et al, 2016[70]	MND	Process	Australia	Systematic review	-	Community, outpatient	To report the prevalence, content, patient/caregiver benefits, HP awareness/support, and healthcare outcomes associated with ACP in MND.
Murray et al, 2016[71]	MND	AD	Australia	Qualitative study	Carers (N=18)	Outpatient	To elicit the experiences of bereaved caregivers of MND patients who had or had not completed the letter of future care.
Perin et al, 2020[55]	Dementia	Process	Italy	Qualitative study	Physicians (N=11)	Outpatient	To explore the perception of physicians providing care to people with dementia

First author, publication year[ref. no.]	Neurodegenerative disorder	ACP definition	Country	Design	Sample	Setting	Aim
							regarding ACP, and to describe their difficulties and needs.
Pettigrew et al, 2019[58]	Dementia	Process/AD	US	Observational, cross-sectional	Carers (N=431)	Outpatient	To deepen understanding of the factors influencing decision making, preferences and plans regarding ACP and EOL care in people with dementia and their caregivers; and to examine differences by race.
Phenwan et al, 2020[49]	Dementia	Process	UK	Literature review	-	Community	To identify factors facilitating or inhibiting ACP/AD initiation and review in people with dementia.<br>To assess the current evidence affecting ACP initiation in people with dementia, so as to inform current policy and practice.
Pollom et al, 2018[77]	Brain tumors	Process	USA	Observational, retrospective study	Patients (N=63)	Outpatient	To describe ACP documentation and referral to palliative care in patients with glioblastoma undergoing radiation therapy.
Preston et al, 2012[72]	MND	EOL planning formal document	UK	Qualitative study	Carers (N=11)	Outpatient	To evaluate the patients' bereaved relatives' experiences of using the Preferred Priorities for Care document and their perceptions about its impact on EOL care.
Rosemond et al, 2017[64]	Dementia	Not reported	US	Qualitative study	Carers (N=16)	NH	To investigate the family members' experiences using goals-of-care decision making of their relatives' death from advanced dementia.
Saevareid et al, 2019[40]	Dementia	Process	Norway	RCT	NHs (N=8)	NH	To improve patient participation in ACP conversations, and to assess patient preferences for treatment and decision making, hopes and worries, documented in the patient health records.
Sampson et al, 2011[37]	Dementia	Process	UK	RCT	Patient-carer dyads (N=32): IG (N=22), CG (N=10).	Outpatient	To assess the feasibility of a two-component intervention to improve EOL care for people with advanced dementia.

First author, publication year[ref. no.]	Neurodegenerative disorder	ACP definition	Country	Design	Sample	Setting	Aim
Seeber et al, 2019[73]	ALS	Process	The Netherlands	Qualitative study	Patients with ALS (N=16); patients with Progressive Muscular Atrophy (N=5)	Outpatient	To evaluate timing and content of discussions on supportive treatment options and EOL care; and to investigate patients' reflections on this practice; and to formulate recommendations about integration of ACP in the patient care.
Sellars et al, 2019[48]	Dementia	Process	Australia	Systematic review	People with dementia (N=389), and their carers (N=1864)	Community	To report the perspectives of people with dementia and their carers on ACP and EOL care in dementia, so as to inform strategies improving patient and carer outcomes.
Song et al, 2016[78]	Brain tumors	Discussions or directive	Australia	Literature review	-	Community, NH, inpatients, outpatient	To systematically evaluate the current evidence and efficacy of ACP in primary brain tumor patients to inform research and clinical practice.
Song et al, 2019[38]	Dementia	Not reported	USA	RCT	Dyads assigned to Spirit in person (N=12); dyads to Spirit remote (N=11).	Outpatient	To adapt and assess feasibility of an ACP intervention for people with dementia and their carers.
Sussman et al, 2020[44]	Dementia	Process	Canada	Qualitative study	People with dementia (N=10), family caregivers (N=8)	community	To investigate the patient and carer perceptions of and experiences with ACP; to explore practices potentially supporting positive engagement with ACP.
Tilburgs et al, 2020[32]	Dementia	Process	The Netherlands	RCT	First workshop: cluster GPs (IG, N=19; CG, N=19). Second workshop: GPs (N=38), people with dementia and carers (IG, N=73; CG, N= 67)	Community, outpatient	To assess the efficacy of an educational intervention aiming to initiate and optimize ACP, with a focus on discussing medical and non-medical preferences of future care.
Tuck et al, 2015[80]	Parkinson's disease	Not reported	USA	Observational, cross-sectional	Patients (N=255)	Outpatient	To identify preferences of patients with Parkinson's disease for timing and initiation of discussions on treatment, prognosis, ACP, and EOL care.
van Rickstal et al, 2019[46]	Dementia	Process	Belgium	Qualitative study	Carers (N=15) of 15 different persons with young-onset dementia.	Outpatient	To assess the extent to which patients, caregivers had already engaged in ACP, and their preferences for engagement in ACP.

First author, publication year[ref. no.]	Neurodegenerative disorder	ACP definition	Country	Design	Sample	Setting	Aim
Van Rickstal et al, 2020[47]	Dementia	Process	Belgium	Qualitative study	Caregivers (N=28)	Outpatient	To explore the carer engagement in and conceptualization of ACP; and identify similarities/differences existing between American and Belgian people with dementia and their carers.
Vandervoort et al, 2014[10]	Dementia	Process	The Netherlands	Observational, cross-sectional	NHs (N=69), with deaths of residents with dementia (N=101).	NH	To assess to what extent AD as verbal spoken advance communication with patient and/or relatives; and the existence of GP orders relate to the quality of dying in people with dementia.
Walter et al, 2019[19]	MS, Parkinson's disease, high grade glioma	Process	The Netherlands	Observational, cross-sectional	Neurologists (N=89) and residents (N=36).	Community	To investigate timing and content of discussions on treatment restrictions and to initiate, withhold, or withdraw treatment in high grade glioma, PD, and MS, from the neurologist's perspective; and to compare the results with international data about ACP in patients with ALS.
Wendrick-van Dael et al, 2020[41]	Dementia	Process/discussion	Belgium	Systematic review	-	-	To investigate the strength of the evidence and provide decision makers with the state of the art on ACP for people with dementia.

ACP, advance care planning; AD, advanced directive; ALS, amyotrophic lateral sclerosis; CG, control group; EOL, end of life care; GP, general practitioner; HP, health professional; IG, intervention group; MND, motor neuron disease; NH, nursing home; RCT, n.a., not applicable; RCT, randomized controlled trial; SDM, shared decision making; UK, United Kingdom.
