# Supplementary material for: Advance Care Planning in Neurodegenerative Disorders: A Scoping Review
Source: Int J Environ Res Public Health. 2022 Jan 12;19(2):803. doi: 10.3390/ijerph19020803 (PMC8775509; doi:10.3390/ijerph19020803)
Supplement: Supplementary file 1 [file ijerph-19-00803-s001.zip › Table S2.pdf]

Table S2. Quality appraisal of the included reviews (AMSTAR-2 tool minus the questions about meta-analysis Q11, Q12, Q15).

First author, publication year[ref. no.]	Q1	Q2	Q3	Q4	Q5	Q6	Q7	Q8	Q9	Q10	Q13	Q14	Q16	Score
Beck et al, 2015[53]	Yes	No	No	Partial yes	No	No	Partial yes	Yes	No	No	No	Yes	Yes	5/13
Geshell et al, 2019[42]	Yes	Partial yes	No	Partial yes	Yes	Yes	No	Yes	Yes	No	Yes	Yes	Yes	9/13
Keijzer-van Laarhoven et al, 2020[54]	Yes	Yes	No	Yes	Yes	Yes	Partial yes	Yes	Yes	No	Yes	No	Yes	9.5/13
Phenwan et al, 2020[49]	Yes	No	No	Yes	Yes	No	No	Partial yes	No	Yes	No	Yes	Yes	6.5/13
Song et al, 2016[78]	Yes	Partial yes	No	Yes	Yes	Yes	Partial yes	Yes	Yes	No	Yes	Yes	Yes	10/13
Lai et al, 2019[43]	Yes	Partial yes	No	Yes	No	No	Partial yes	No	Yes	No	Yes	Yes	Yes	7/13
Murray et al, 2016[70]	Yes	Partial yes	No	Partial yes	No	No	Partial yes	Yes	Yes	Yes	Yes	Yes	No	7/13
Sellars et al, 2019[48]	Yes	Partial yes	Yes	Partial yes	Yes	No	Partial yes	Partial yes	Yes	No	Yes	Yes	Yes	9/13
Wendrich-van Dael et al, 2020	Yes	Yes	No	Partial yes	No	No	Partial yes	Yes	Yes	No	Yes	Yes	Yes	8/13

QUESTIONS

1. Did the research questions and inclusion criteria include the components of PICO?
2. Did the report of the review contain an explicit statement that the review methods were established prior to the conduct of the review and did the report justify any significant deviations from the protocol?
3. Did the review authors explain their selection of the study designs for inclusion in the review?
4. Did the review authors use a comprehensive literature search strategy?
5. Did the review authors perform study selection in duplicate?
6. Did the review authors perform data extraction in duplicate?
7. Did the review authors provide a list of excluded studies and justify the exclusions?
8. Did the review authors describe the included studies in adequate detail?
9. Did the review authors use a satisfactory technique for assessing quality in individual studies that were included in the review?
10. Did the review authors report on the sources of funding for the studies included in the review?
13. Did the review authors account for quality in individual studies when interpreting/discussing the results of the review?
14. Did the review authors provide a satisfactory explanation for, and discussion of, any heterogeneity observed in the results of the review?
16. Did the review authors report any potential sources of conflict of interest, including any funding they received for conducting the review?
