# Supplementary material for: Advance Care Planning in Neurodegenerative Disorders: A Scoping Review
Source: Int J Environ Res Public Health. 2022 Jan 12;19(2):803. doi: 10.3390/ijerph19020803 (PMC8775509; doi:10.3390/ijerph19020803)
Supplement: Supplementary file 1 [file ijerph-19-00803-s001.zip › Table S3s.pdf]

Table S3. CASP quality assessment for case control studies.

**First author, publication year[ref. no.]**	1. Did the study address a clearly focused issue?	2. Did the authors use an appropriate method to answer their question?	3. Were the cases recruited in an acceptable way?	4. Were the controls selected in an acceptable way?	5. Was the exposure accurately measured to minimize bias?	6. Have the authors taken account of the potential confounding factors in the design and/or in their analysis?	7. How precise was the estimate of the treatment effect?	8. Do you believe the results?	Score
Ampe et al, 2017[61]	Y	Y	Y	U	Y	U	U	Y	5/8
Cotter et al, 2019[62]	Y	Y	U	U	U	U	Y	N	3/8

Y= yes; N= no; U= unclear
