# Supplementary material for: Advance Care Planning in Neurodegenerative Disorders: A Scoping Review
Source: Int J Environ Res Public Health. 2022 Jan 12;19(2):803. doi: 10.3390/ijerph19020803 (PMC8775509; doi:10.3390/ijerph19020803)
Supplement: Supplementary file 1 [file ijerph-19-00803-s001.zip › Table S4.pdf]

Table S4. CASP quality assessment for cohort studies.

**First author, publication year**[ref. no]	1. Did the study address a clearly focused issue?	2. Was the cohort recruited in an acceptable way?	3. Was the exposure accurately measured to minimise bias?	4.Was the outcome accurately measured to minimise bias?	5.a Have the authors identified all important confounding factors?<br><br>5.b Have they taken account of the confounding factors in the design and/or analysis?	6.a Was the follow up of subjects complete enough?<br><br>6.b Was the follow up of subjects long enough?	7. How precise are the results?	8. Do you believe in the results	Score
Cheung et al, 2017[82]	Y	Y	Y	Y	U	N/A	U	Y	5/8
Goossens et al, 2020[51]	Y	Y	Y	Y	U	N/A	U	Y	5/8
Hossler et al, 2011[68]	Y	Y	Y	U	U	N/A	U	Y	4/8
Huang et al, 2020[63]	Y	Y	Y	Y	U	N/A	Y	Y	6/8
Katwa et al, 2020[60]	Y	U	N/A	U	U	N/A	U	Y	2/8
Konttila et al, 2020[57]	Y	Y	Y	Y	Y	N/A	Y	Y	7/8
Lee et al, 2018[56]	Y	Y	Y	Y	U	N/A	Y	Y	6/8
Levi et al, 2017[69]	Y	Y	Y	U	Y	N/A	Y	Y	6/8
Moss et al, 2018[59]	Y	U	U	U	U	N/A	U	Y	2/8
Pettigrew et al, 2019[58]	Y	Y	Y	Y	Y	N/A	Y	Y	7/8

**First author, publication year**[ref. no]	1. Did the study address a clearly focused issue?	2. Was the cohort recruited in an acceptable way?	3. Was the exposure accurately measured to minimise bias?	4.Was the outcome accurately measured to minimise bias?	5.a Have the authors identified all important confounding factors?<br><br>5.b Have they taken account of the confounding factors in the design and/or analysis?	6.a Was the follow up of subjects complete enough?<br><br>6.b Was the follow up of subjects long enough?	7. How precise are the results?	8. Do you believe in the results	Score
Pollom et al, 2018[77]	Y	Y	U	U	U	N/A	Y	Y	3/8
Tuck et al, 2015[80]	Y	Y	Y	Y	U	N/A	U	Y	5/8
Vandervoort et al, 2014[10]	Y	Y	U	U	Y	N/A	Y	Y	5/8
Walter et al, 2019[19]	Y	Y	Y	Y	Y	N/A	U	U	5/8

Y= yes; N= no; N/A= not applicable; U= unknown.
