# Supplementary material for: Advance Care Planning in Neurodegenerative Disorders: A Scoping Review
Source: Int J Environ Res Public Health. 2022 Jan 12;19(2):803. doi: 10.3390/ijerph19020803 (PMC8775509; doi:10.3390/ijerph19020803)
Supplement: Supplementary file 1 [file ijerph-19-00803-s001.zip › Table S5.pdf]

Table S5. CASP quality assessment for qualitative studies.

**First author, publication year[ref. no.]**	1. Was there a clear statement of the aims of the research?	2. Is a qualitative methodology appropriate?	3. Was the research design appropriate to address the aims of the research?	4. Was the recruitment strategy appropriate to the aims of the research?	5. Was the data collected in a way that addressed the research issue?	6. Has the relationship between researcher and participants been adequately considered?	7. Have ethical issues been taken into consideration?	8. Was the data analysis sufficiently rigorous?	9. Is there a clear statement of findings?	Score
Abbott et al, 2017[81]	Y	Y	Y	Y	Y	U	Y	Y	Y	8/9
Cutshall et al, 2020[74]	Y	Y	Y	Y	Y	U	N	U	Y	6/9
Fried et al, 2020[45]	Y	Y	Y	Y	Y	U	Y	Y	U	7/9
Fritz et al, 2020[75]	Y	Y	U	Y	Y	U	Y	U	Y	6/9
Llewellyn et al, 2018[76]	Y	Y	U	Y	Y	U	Y	Y	Y	7/9
Lum et al, 2019[9]	Y	Y	U	Y	Y	U	Y	Y	Y	7/9
Murray et al, 2016[71]	Y	Y	U	Y	Y	Y	Y	Y	Y	8/9
Perin et al, 2020[55]	Y	Y	Y	Y	Y	U	Y	Y	Y	8/9
Preston et al, 2012[72]	Y	Y	U	Y	Y	U	N	Y	Y	6/9
Rosemond et al, 2017[64]	Y	Y	Y	Y	Y	U	Y	Y	Y	8/9
Seeber et al, 2019[73]	Y	Y	Y	Y	Y	U	Y	Y	Y	8/9
Sussman et al, 2020[44]	Y	Y	Y	Y	Y	U	Y	Y	Y	8/9
Van Rickstal et al, 2019[46]	Y	Y	Y	U	Y	U	Y	Y	Y	7/9
Van Rickstal et al, 2020[47]	Y	Y	Y	Y	Y	U	Y	Y	Y	8/9

Y= yes; N= no; U= unknown.
