# Supplementary material for: Advance Care Planning in Neurodegenerative Disorders: A Scoping Review
Source: Int J Environ Res Public Health. 2022 Jan 12;19(2):803. doi: 10.3390/ijerph19020803 (PMC8775509; doi:10.3390/ijerph19020803)
Supplement: Supplementary file 1 [file ijerph-19-00803-s001.zip › Table S6.pdf]

Table S6. Mixed Methods Appraisal Tool (MMAT) for mixed method studies.

**First author, publication year[ref. no.]**	Are there clear research questions?	1	Do the collected data allow to address the research questions?	2	Is there an adequate rationale for using a mixed methods design to address the research question?	3	Are the different components of the study effectively integrated to answer the research question?	4	Are the outputs of the integration of qualitative and quantitative components adequately interpreted?	5	Are divergences and inconsistencies between quantitative and qualitative results adequately addressed?	6	Do the different components of the study adhere to the quality criteria of each tradition of the methods involved?	7	Score
Dassel et al, 2019[52]	Y	**Y**	Y	**Y**	Y	**Y**	Y	**Y**	Y	**Y**	U	**U**	Y	**Y**	6/7
Lee et al, 2020[50]	Y	**Y**	Y	**Y**	Y	**Y**	Y	**Y**	Y	**Y**	U	**U**	U	**U**	5/7
