# Supplementary figures and images for: Advance Care Planning in Neurodegenerative Disorders: A Scoping Review
Source: Int J Environ Res Public Health. 2022 Jan 12;19(2):803. doi: 10.3390/ijerph19020803 (PMC8775509; doi:10.3390/ijerph19020803)

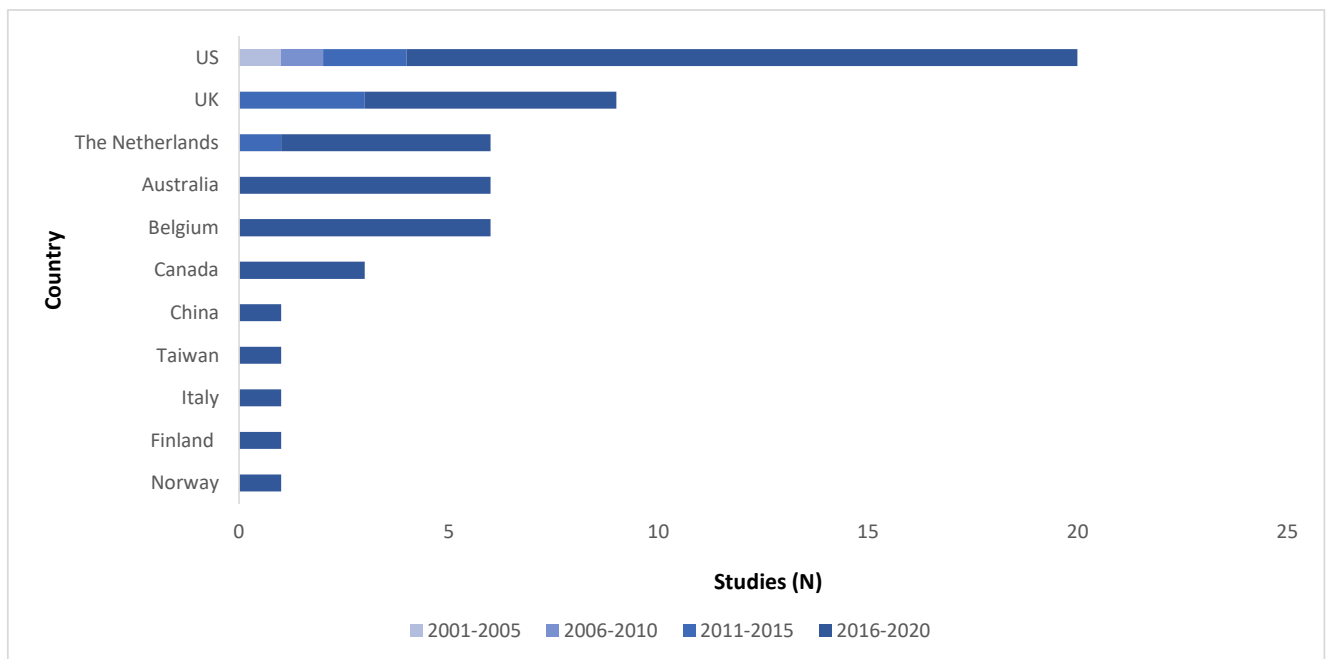

Supplement: Supplementary file 1 [file ijerph-19-00803-s001.zip › Fig. S1.pdf]
